# From Organotypic Mouse Brain Slices to Human Alzheimer Plasma Biomarkers: A Focus on Microglia

**DOI:** 10.3390/biom14091109

**Published:** 2024-09-03

**Authors:** Katharina Steiner, Sakir Necat Yilmaz, Alessa Gern, Josef Marksteiner, Klaus Faserl, Mathias Villunger, Bettina Sarg, Christian Humpel

**Affiliations:** 1 Laboratory of Psychiatry and Experimental Alzheimer’s Research, Medical University of Innsbruck, 6020 Innsbruck, Austria; k.steiner@i-med.ac.at (K.S.); nyilmaz@mersin.edu.tr (S.N.Y.); alessagern00@gmail.com (A.G.); 2 Department of Histology and Embryology, Faculty of Medicine, Mersin University, Mersin 33110, Turkey; 3 Department of Psychiatry and Psychotherapy A, Hall State Hospital, 6060 Hall in Tirol, Austria; josef.marksteiner@tirol-kliniken.at; 4 Protein Core Facility, Institute of Medical Biochemistry, CCB-Biocenter, Medical University of Innsbruck, 6020 Innsbruck, Austria; klaus.faserl@i-med.ac.at (K.F.); mathias.villunger@i-med.ac.at (M.V.); bettina.sarg@i-med.ac.at (B.S.)

**Keywords:** Alzheimer’s disease, biomarker, plasma, organotypic brain slice, microglia, microcontact printing, mass spectrometry

## Abstract

Alzheimer’s disease is a severe neurodegenerative disorder, and the discovery of biomarkers is crucial for early diagnosis. While the analysis of biomarkers in cerebrospinal fluid is well accepted, there are currently no blood biomarkers available. Our research focuses on identifying novel plasma biomarkers for Alzheimer’s disease. To achieve this, we employed a technique that involves coupling human plasma to mouse organotypic brain slices via microcontact prints. After culturing for two weeks, we assessed Iba1-immunopositive microglia on these microcontact prints. We hypothesized that plasma from Alzheimer’s patients contains factors that affect microglial migration. Our data indicated that plasma from Alzheimer’s patients significantly inhibited the migration of round Iba1-immunoreactive microglia (13 ± 3, *n* = 24, *p* = 0.01) compared to healthy controls (50 ± 16, *n* = 23). Based on these findings, we selected the most promising plasma samples and conducted mass spectrometry using a differential approach, and we identified four potential biomarkers: mannose-binding protein C, macrophage receptor MARCO, complement factor H-related protein-3, and C-reactive protein. Our method represents a novel and innovative approach to translate research findings from mouse models to human applications.

## 1. Introduction

### 1.1. Biomarkers in Alzheimer’s Disease

Clinical evaluation remains the most frequently used method for diagnosing Alzheimer’s disease (AD). Laboratory tests and imaging techniques also aid in diagnosis. Despite advancements in diagnostic tools, the disease is often diagnosed in advanced stages or postmortem through the observation of beta-amyloid (Aβ) plaques and tau/neurofibrillary tangles in brain tissue. While there is widespread agreement that some biomarkers change long before clinical symptoms appear, there is no consensus on what these biomarkers are. Early diagnosis is thought to facilitate treatment of the disease before it progresses significantly. Starting treatment before irreversible neuron loss occurs is crucial. For diagnosing AD or its early forms, such as mild cognitive impairment (MCI), identifying biomarkers in human fluids is essential. There is general agreement on four well-defined and validated cerebrospinal fluid (CSF) biomarkers in AD: Aβ-42, Aβ-40, total tau, and phosphorylated (phospho-)tau-181 [1]. However, routinely usable biomarkers in other biological samples, such as plasma, have not yet been identified.

### 1.2. Plasma Biomarkers in Alzheimer’s Disease

Research on plasma biomarkers for AD has a long history and encompasses a substantial body of work. Some authors have identified a panel of 18 biomarkers in plasma [2], but this finding has not been reproducible [3]. A Japanese group identified the ratio between amyloid precursor protein (APP) and Aβ using mass spectrometry, a method that is both time-consuming and costly [4]. Unfortunately, Aβ plasma biomarkers do not provide consistent results for diagnosing AD. To date, there are no plasma biomarkers available for diagnosing AD in humans, but extensive research is being conducted [5]. There is hope that plasma phospho-tau proteins (−181 or −217) may become reliable biomarkers [6,7]. Although Aβ and tau proteins and their derivatives are the most commonly studied biomarkers, other candidates have also been examined, including lipids, metabolites, vitamins, inflammatory molecules and cytokines, non-coding RNAs, oxidative stress markers, gut-microbiome-derived molecules, and anti-beta-amyloid antibodies [8,9,10,11,12,13]. Plasma biomarkers would greatly simplify diagnosis concerning low costs, non-invasive intervention, and large-scale feasibility, compared to CSF or neuroimaging [5]. There is a clear need to discover new biomarkers in plasma or other human fluids, such as saliva, to enable earlier diagnosis and symptomatic treatment of AD or other forms of dementia [14,15].

### 1.3. Microglia in Alzheimer’s Disease

Under healthy conditions, microglial cells protect brain tissue from pathogens and maintain homeostasis around neurons. They exhibit a ramified morphology when quiescent but transform into an amoeboid, round shape when activated [16]. In AD, microglia initially play a protective role by eliminating the toxic accumulations of Aβ through phagocytosis. However, when activated, they can contribute to AD by releasing inflammatory factors and damaging neuronal synapses, leading to neurodegeneration and neuron loss [17]. Prolonged activation can drive microglia into a senescent, dystrophic state, diminishing their activity in the late stages of the disease [18]. Microglia contribute to the brain’s complex inflammatory response by modulating various inflammatory factors, such as IL-1α, IL-1β, IL-6, IL-8, IL-12, IL-18, and IL-23, IFN-γ, TNF-α, granulocyte-macrophage colony-stimulating factor (GM-CSF) [19,20], monocyte chemotactic protein 1 (MCP-1), MCP-113, fractalkine [21,22], chemoattractant proteins, prostaglandins, complement factors, thromboxanes, pentraxins, nitric oxide (NO), reactive oxygen species, leukotrienes, proteases, protease inhibitors, adhesion molecules [23], coagulation factors, and C-reactive protein [24,25,26]. In addition to their pro-inflammatory properties, microglia also have anti-inflammatory functions and can produce regulatory cytokines, such as IL-10 and transforming growth factor-β (TGF-β) [27]. This complex role of microglia in the AD brain allows for the exploration of biomarkers linked to both microglia and pro-/anti-inflammatory molecules.

### 1.4. Organotypic Brain Slices as a Tool to Search for Biomarkers

Organotypic brain slices are derived from postnatal days 8–10 mice and preserve the brain’s complex three-dimensional architecture and microenvironment when cultured, as extensively reviewed by Humpel [28]. These slices can remain viable for extended periods, ranging from several weeks to months, making them an excellent ex vivo model, closely resembling in vivo conditions. In the past, molecules studied in brain slices were introduced either through the culture medium or by adsorbing them onto various biomaterials. In our laboratory, we have developed an innovative technique utilizing organotypic brain slices combined with microcontact printing, through which different molecules are deposited in spaced lines at the micrometer scale using collagen as a scaffold. This technique is detailed in the review by Steiner and Humpel [29]. Using this method, we have investigated the activation of microglia [30] and blood vessels [31], as well as the effects of nerve growth factor (NGF) on cholinergic neurons [32]. Microcontact printing allows precise printing of proteins and peptides of interest onto semipermeable membranes, facilitating their interaction with organotypic brain slices in a controlled manner. This interaction between the microcontact print (µCP) and organotypic brain slices activates brain cells within the slice, promoting their migration and differentiation along the microcontact-printed lines.

In our current study, we aim to use microcontact printing for the first time to apply plasma from human patients and observe its effects on the migration of activated microglia. We hypothesize that plasma from AD patients may contain or lack factors compared to healthy subjects, which could differentially influence the activation and migration of Iba1-immunoreactive microglia in mouse brain slices. We utilize mass spectrometry on the most promising samples to identify new potential biomarkers.

## 2. Materials and Methods

### 2.1. Human Plasma Samples

For this study, human plasma samples were collected from three groups: healthy controls, patients diagnosed with MCI, and patients diagnosed with AD. All participants were aged above 60 years. The study protocol was approved by the local ethics committee of Innsbruck Medical University (AN2015.0159 351/4.7 405/5.5 (4484a), granted to Prim. Univ.-Prof. Dr. Josef Marksteiner) and adhered to the principles of the Helsinki Declaration. Written informed consent was obtained from all subjects. Patients were recruited from Landeskrankenhaus Hall/Tirol, Austria, under the supervision of Prim. Univ.-Prof. Dr. Josef Marksteiner. Plasma samples were collected since 2014 and have been stored at −80 °C without thawing cycles. To minimize degradation effects, samples from all three groups were uniformly analyzed from samples collected in the same year.

**Figure 1:**
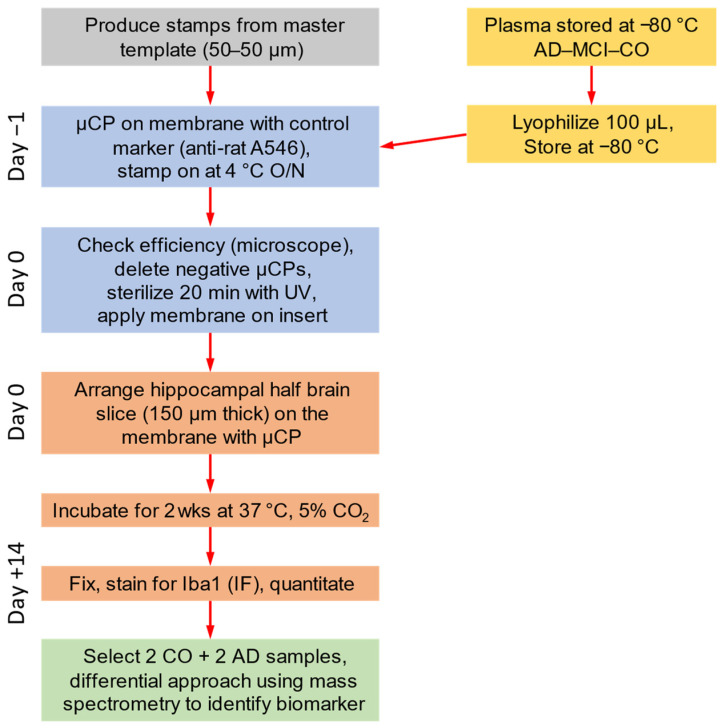
Experimental design to identify human plasma biomarkers. The stamps made of polydimethylsiloxane had 800 µm-long lines, each with a width of 50 µm and spaced 50 µm apart (50–50 µm, grey). Plasma is collected from patients with Alzheimer’s disease (AD), mild cognitive impairment (MCI), and healthy controls (CO) and stored at −80 °C (yellow). The lyophilized plasma (yellow) is microcontact-printed (µCP) on membranes one day before brain slice preparation and stored with stamp on at 4 °C overnight (O/N, blue). After printing efficiency control and sterilization with UV light (blue), microcontact prints (µCPs) are connected to mouse organotypic half-brain slices of the hippocampus and cultured for two weeks (orange). The brain slices are then fixed and immunofluorescently (IF) stained for the microglial marker Iba1 for quantification (orange). Selected samples are processed for mass spectrometry analysis (green).

The patients’ diagnoses have been extensively detailed in previous publications [33]. For this study, only these plasma samples were used. Briefly, all subjects underwent a comprehensive neuropsychological assessment, including the Geriatric Depression Scale (GDS) and the Mini-Mental State Examination (MMSE). Only patients meeting diagnostic criteria for MCI and AD were included. Structural magnetic resonance imaging (MRI) was conducted using a 1.5 Tesla Siemens Symphony MRI scanner for all patients. Participants underwent continuous statin or ezetimibe treatment for at least 3 months before study entry. No patient had a cholesterol level > 240 g/dL that was not treated with a statin or ezetimibe. Particular care was taken to exclude subjects with severe medical or neurological conditions that could account for cognitive deficits. Following group assignment, 10 mL of EDTA blood was collected from each patient and processed within 24 h. The samples underwent centrifugation (2300× *g*, 5 min), and the plasma was immediately frozen at −80 °C (Figure 1).

### 2.2. Microcontact Printing of Human Plasma

The process of microcontact printing using collagen hydrogel has been extensively detailed in our methodological review [29]. The master template, purchased from GeSiM (www.gesim.de, accessed on 27 August 2024, 01454 Radeberg, Germany), featured 50 lines spaced at 50 µm intervals, with a width of 50 µm and a length of 800 µm (Figure 1). The stamps were fabricated using polydimethylsiloxane (PDMS; Sylgard 184 Silicone Elastomer Kit, Dow, Seneffe, Belgium, 01673921), as outlined in our methodological review [29]. For microcontact printing of plasma, the following steps were taken to adapt the protocol: First, 100 µL of plasma was lyophilized (Figure 1) and subsequently resuspended in 67 µL of bovine collagen solution type I (stock solution of 3 mg/mL, Sigma-Aldrich, St. Louis, MO, USA, 804592). Then, 10 µL of 100 mM phosphate-buffered saline (PBS; pH 7.4, autoclaved, 10 mM Na_2_HPO_4_ (Merck Millipore, Darmstadt, Germany, 106586), 137.75 mM NaCl (Roth, Karlsruhe, Germany, 3957.1), and 2.68 mM KCl (Roth, Karlsruhe, Germany, 6781.3)) and 5 µL of 10 mM PBS were added. To assess the loading efficiency, 5 µL of red-fluorescent Alexa Flour 546 anti-rat (Invitrogen, Thermo Fisher Scientific, Waltham, MA, USA, A11081) was included. NaOH (1N, Roth, Karlsruhe, Germany, 6771.3) was added to adjust the pH to 7.2. Last, 12.5 µL of the crosslinker 4-arm-poly(ethylene glycole) (PEG) succinimidyl succinate (stock solution of 12.5 mg/mL, Sigma-Aldrich, St. Louis, MO, USA, JKA7006) was added.

The solution was evenly spread across the stamp by placing a coverslip on top. After a 15 min incubation at 37 °C, the coverslip was removed and utilized to wipe away any excess solution. Once the stamp had completely dried, it was inverted, and the solution was transferred onto a semipermeable extra membrane (Isopore, Merck Millipore, Darmstadt, Germany, HTTP02500) by applying pressure with 18 g coins for 60 min at room temperature in the dark. After removing the coins, the assembly was stored overnight at 4 °C. The following day, the stamp was detached from the extra membrane, and the printing efficiency was assessed under a microscope. Membranes that did not show adequate microcontact printing were discarded (Figure 1). A small dot of permanent marker was used to indicate the print position for later alignment with brain slices. The membranes were exposed to UV light for 20 min for sterilization and subsequently equilibrated with sterile medium (Figure 1).

To confirm that recombinant mouse MCP-1 protein (MedChemExpress, Monmouth Junction, NJ, USA, HY-P7764) was microcontact-printed onto the membranes, the print was stained using immunofluorescence. Briefly, membranes were fixed in 4% paraformaldehyde (PFA) for 30 min immediately after microcontact printing. After a quick rinse in 10 mM PBS, the membranes were incubated in PBS + bovine serum albumin (BSA) 0.2% with primary antibody against MCP-1 (Proteintech, Rosemont, IL, USA, 26161-1-AP, 1:500) overnight. This was followed by washing and detection with an anti-rabbit Alexa Fluor 488 secondary antibody (Invitrogen, Thermo Fisher Scientific, Waltham, MA, USA, A21206, 1:400).

### 2.3. Organotypic Brain Slices

Organotypic half-brain slices were prepared, as previously described in detail by us [29]. Briefly, C57BL/6 wild-type mice at postnatal days 8–10 were rapidly sacrificed, and the brains were dissected and affixed onto the sample holder platform with an adhesive (Loctite 401, Henkel, Düsseldorf, Germany 231435) under sterile conditions. Coronal brain slices, each 150 µm-thick and taken from the hippocampus region, were cut in sterile medium using a water-cooled vibratome (Leica, Nussloch, Germany, VT1000S). Once horizontally bisected, the upper section containing the hippocampus was transferred onto microcontact-printed 0.4 µm semipermeable extra membranes (Isopore, Merck Millipore, Darmstadt, Germany, HTTP02500) in cell culture inserts (Millicell, Merck Millipore, Darmstadt, Germany, PICM03050) (Figure 1). The exact composition of the brain slice and µCP was extensively reviewed elsewhere by us [29]. Briefly, the microcontact-printed lines are vertical and run parallel to each other. The half-brain slice is positioned atop the µCP, covering the µCP’s upper portion. The microcontact-printed lines extend downward from the base of the brain slice.

Each well of the six-well plate (Sarstedt, Nümbrecht, Germany, 83.3920) was filled with 1 mL of medium (pH 7.2, sterile filtrated, 1× MEM (Gibco, Thermo Fisher Scientific, Waltham, MA, USA, 11012044), 5.12 mM NaHCO_3_ (Merck Millipore, Darmstadt, Germany, 106329), 31.5 mM glucose (Merck Millipore, Darmstadt, Germany, 49159), 2 mM glutamine (Merck Millipore, Darmstadt, Germany, 100289), 10% heat-inactivated horse serum (HS, Gibco, Thermo Fisher Scientific, Waltham, MA, USA, 16050-122), 0.25 × HBSS (Gibco, Thermo Fisher Scientific, Waltham, MA, USA, 24020091), and 1× antibiotic-antimitotic solution (Sigma-Aldrich, St. Louis, MO, USA, A5955)). The brain slices were cultured at 37 °C with 5% CO_2_ for 2 weeks, with medium changes occurring weekly (Figure 1). Following the culture period, the slices were fixed in 4% PFA for 3 h at 4 °C, and then stored in 10 mM PBS with 0.1% NaN_3_ at 4 °C until further use.

All experiments complied with Austrian regulations for the ethical use of animals and adhered to the ethical principles of the three Rs (replace, reduce, and refine) to minimize animal use. In accordance with Austrian laws, all animal experiments were categorized as “Organentnahme”.

### 2.4. Immunofluorescence for Microglia

Immunostaining was performed as previously described elsewhere [29]. Briefly, the fixed brain slices on membranes were incubated in 0.1% Triton-PBS (T-PBS) for 30 min at room temperature with gentle shaking. Then, the brain slices were washed for 3 × 3 min each with 10 mM PBS and subsequently blocked in T-PBS 0.1% + BSA 0.2% + HS 20% for 30 min with gentle shaking. Following blocking, the brain slices were incubated in T-PBS 0.1% + BSA 0.2% with primary antibodies against microglial Iba1 (Fujifilm Wako Pure Chemical Corporation, Osaka, Japan, 019-19741; 1:500) at 4 °C for 48 h. After washing again for 3 × 3 min with PBS, the sections were incubated with anti-rabbit green-fluorescent Alexa Fluor 488 (Invitrogen, Thermo Fisher Scientific, Waltham, MA, USA, A21206, 1:400) and blue-fluorescent nuclear dye DAPI (Sigma-Aldrich, St. Louis, MO, USA, D9542, 1:10,000) in T-PBS 0.1% + BSA 0.2% at RT for 1 h with gentle shaking and protected from light. Finally, the brain slices were washed again for 3 × 3 min with PBS before mounting on glass slides with Mowiol. Alexa Fluor 488 was observed in the green channel (ex 480/40 nm and em 527/30 nm) using a fluorescence microscope (Olympus, Tokyo, Japan, BX61) and connected Openlab software (version 5.5.0, Improvision, Coventry, UK).

### 2.5. Mass Spectrometry

Sample preparation: Fourteen highly abundant plasma proteins were depleted using High Select™ Depletion Spin Columns (P/N: A36369, Fisher Scientific, Vienna, Austria), according to the manufacturer’s instructions. Proteins in the flow-through (400 µL) were reduced with 40 µL of 100 mM dithiothreitol in PBS buffer for 30 min at 56 °C, followed by alkylation of free cysteines with 40 µL of 550 mM iodacetamide in PBS buffer for 20 min at room temperature in the dark. Samples were lyophilized to 200 µL and diluted by a factor of 5 with acetonitrile, vortexed, and centrifuged for 5 min at 16,000× *g*. The pellet was washed twice with ethanol and dissolved in 100 µL of 100 mM TEAB buffer (pH 8.5). Proteins were digested with 1 µg of trypsin (Sequencing Grade Modified Trypsin, P/N: V5111, Promega, Walldorf, Germany) overnight at 37 °C under agitation. Digested peptides were subjected to TMT labeling (TMT10plex™ Label Reagent Set, P/N A58332, Fisher Scientific, Vienna, Austria), according to the manufacturer’s instruction. All samples were pooled, lyophilized to dry, and re-dissolved in 85 µL of 0.1% formic acid. Peptides were then high-pH-fractionated by reversed-phase chromatography using a XBridge Peptide BEH C18 column (4.6 mm × 250 mm, 300 Å, 5 µm; P/N 186003625, Waters), according to [34].

Liquid chromatography was coupled to tandem mass spectrometry (nano-LC-MS) and is described in brief: Peptide digests were analyzed using an UltiMate 3000 nano-HPLC system coupled to an Orbitrap Eclipse mass spectrometer (Fisher Scientific, Vienna, Austria), as described previously [35]. In brief, peptides were separated on a homemade column (100 μm i.d. × 17 cm length) packed with 2.4 μm of C18 material (Reprosil, Dr. A. Maisch HPLC GmbH) using an acetonitrile gradient with a total gradient time of 142 min. The Orbitrap Eclipse mass spectrometer operated in the data-dependent mode with a cycle time of 3 s. The Survey full-scan MS spectra were acquired at a resolution of 120,000 and the MS2 spectra at 50,000. The fragmentation was performed by higher-energy collisional dissociation using a normalized collision energy of 38.

MS Database search: Proteome Discoverer (version 3.1, Fisher Scientific, Vienna, Austria) was used to process the MS data, and the MS/MS spectra were compared with the Sequest HT engine against a Uniprot human reference proteome database (last modified 27 March 2024). We used the following search parameters: (a) enzyme specificity was set to trypsin, with 2 missed cleavages allowed, (b) fixed modification was carbamidomethyl on cysteine, (c) variable modifications were oxidation of methionine, (d) precursor mass tolerance was set to 10 ppm, (e) fragment mass tolerance was 20 mmu, and (f) the maximum false discovery rate (FDR) for protein and peptide identification was set to 1%. For the quantitative analysis, protein fold changes were calculated (using TMTpro reporter ion intensities present in MS2 scans). The different sequence similarities between humans and mice were compared (NCBI BLAST+ tool of the EMBL’s European Bioinformatics Institute [36]).

### 2.6. Data Analysis and Statistics

Quantitative analysis was conducted in a blinded manner. Only microglia that had clearly migrated out from the brain slice and were distinctly positioned on the microcontact-printed lanes were included in the quantitative analysis. The number of Iba1-immunoreactive migrated microglia (round, ramified, and macrophagic) was assessed along the boundary of the horizontal cutting side over a length of 300 µm (or 6 microcontact-printed lanes) using the manual cell counter of ImageJ software (version 1.48; National Institute of Health, Bethesda, MD, USA). To measure the migration distance, the pixel-to-µm ratio was calibrated based on microscope measurements, and the coordinates of each counted microglia were averaged per brain slice. The sample size (*n*) indicates the number of independent animals used or the number of plasma µCPs analyzed from different patients. All data are presented as mean ± standard error of the mean (SEM). Statistical analysis was performed using one-way ANOVA followed by a Fisher LSD post hoc test, with *p* < 0.05 considered significant.

## 3. Results

### 3.1. Efficiency of Plasma Microcontact Printing

**Figure 2:**
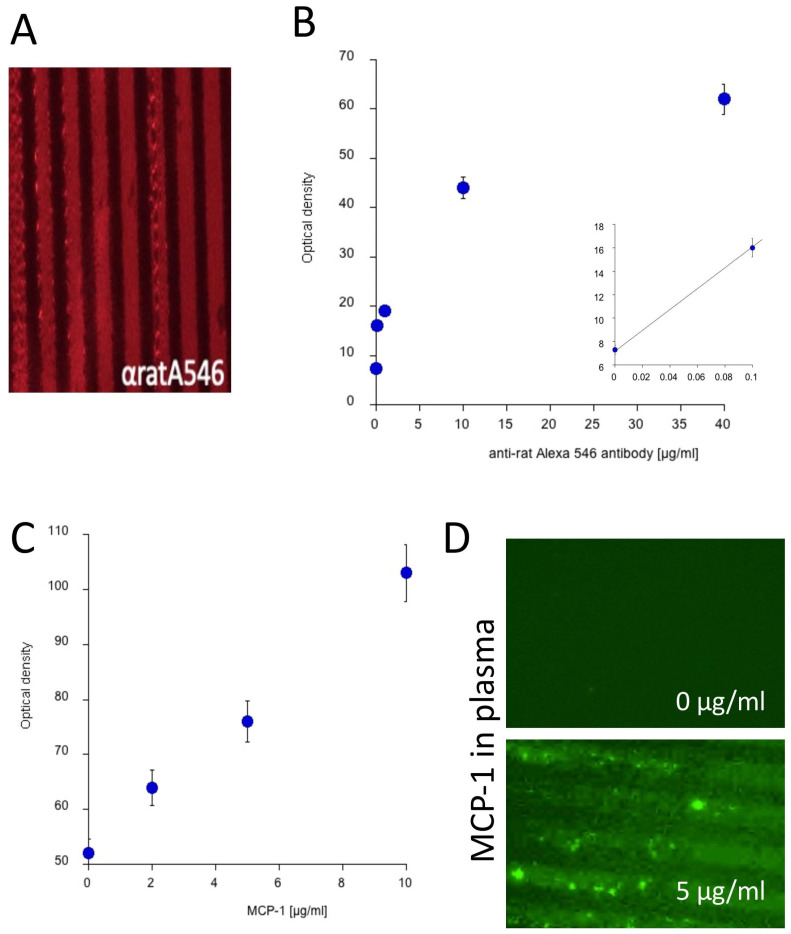
Efficiency of plasma microcontact printing was assessed using monocyte chemoattractant protein 1 (MCP-1) protein. (**A**) A red-fluorescent anti-rat Alexa Fluor 546 antibody served as a control, displaying distinct red lanes. (**B**) The optimal results were achieved with 10 µg/mL of antibody per application. (**C**) Printing different concentrations of MCP-1 (0 to 10 µg/mL) revealed that already, 1 µg/mL of MCP-1 was visible in the print on the membranes. (**D**) Spiking healthy control plasma with 5 µg/mL of MCP-1, followed by microcontact printing and detection via immunofluorescence, clearly revealed MCP-1-specific microcontact-printed lanes compared to the control without MCP-1 in the plasma.

To assess the microcontact printing efficiency, a red-fluorescent anti-rat Alexa Fluor 546 antibody was added to the collagen hydrogel solution, microcontact-printed, and evaluated microscopically. Efficient microcontact printing was achieved, as evidenced by multiple red-fluorescent lanes (Figure 2A). A dose–response curve showed that 0.1 µg/mL of the antibody was effective for visualizing µCPs, whereas 35 µg/mL did not further improve the visualization, revealing an optimal antibody concentration of 10 µg/mL (Figure 2B). To determine the minimal detectable protein concentration in µCPs, MCP-1 ranging from 0 to 10 µg/mL was loaded and subsequently identified via immunofluorescence. Results demonstrated that already, 1 µg/mL of MCP-1 could be visualized in the print on the membrane (Figure 2C). Additionally, spiking 5 µg/mL of MCP-1 into healthy control plasma revealed distinct bands and confirmed successful protein microcontact printing (Figure 2D).

### 3.2. Iba1-Postive Microglial Immunoreactivity

**Figure 3:**
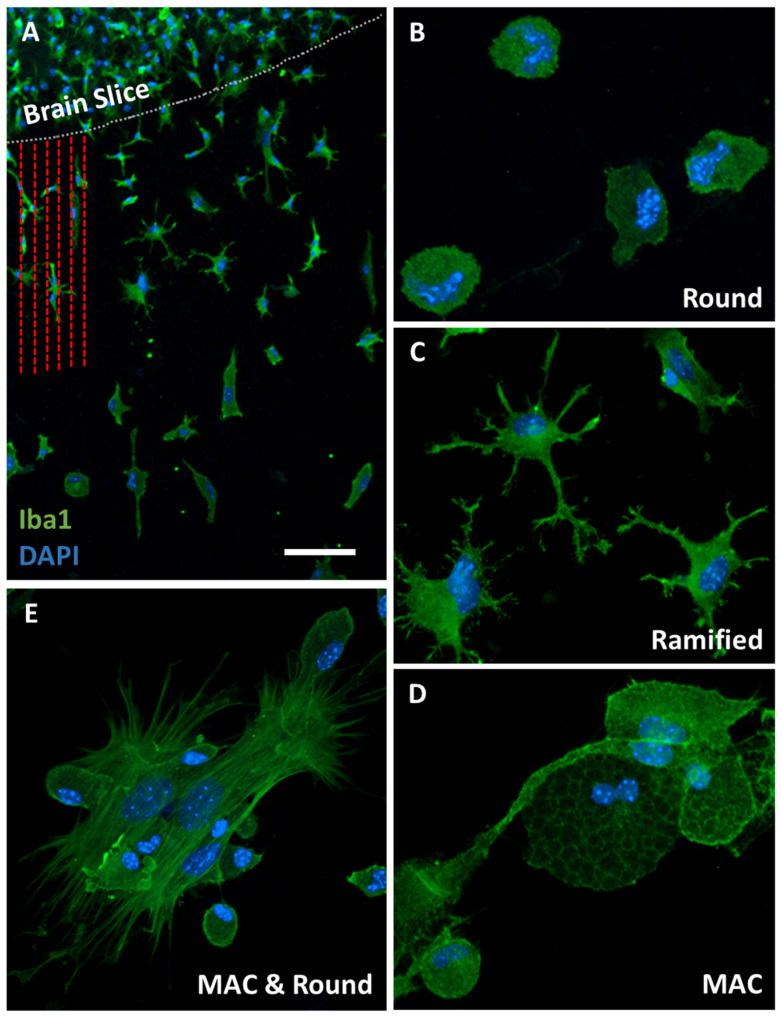
Immunofluorescence staining for Iba1-positive microglia. Microglia were stained by immunofluorescence using Alexa Fluor 488 (green) and counterstained with nuclear DAPI (blue). (**A**) Brain slice (border indicated with the white dotted line) with several migrated microglia along the microcontact-printed lanes (red dashed lines). (**B**–**D**) We identified three types of migrated microglia: small round (**B**), ramified with many processes (**C**), and large macrophagic (MAC) microglia (**D**). (**E**) Large macrophagic microglia are compared with small round microglia. Scale bar = 250 µm (**A**) and 50 µm (**B**–**E**).

Microglia were immunofluorescently stained using the well-established microglial marker Iba1. Immunofluorescence revealed several Iba1-positive microglia that had migrated out of the brain slice along the microcontact-printed lanes (Figure 3A). DAPI staining was utilized to visualize the nuclei of these cells (Figure 3A). We identified three types of migrated and activated Iba1-positive microglia: small round microglia (Figure 3B), highly ramified microglia with numerous processes (Figure 3C), and large macrophagic microglia (Figure 3D,E).

### 3.3. Effects of MCP-1 on Microglial Migration

To evaluate the activation of Iba1-positive microglia, brain slices were treated with MCP-1, a well-known microglial activator. When slices were incubated without MCP-1, nearly no microglia were activated. However, the presence of MCP-1 in the medium significantly increased the number of migrated round microglia, ramified microglia, and macrophagic microglia (Table 1). Microcontact printing of collagen further enhanced the migration of Iba1-positive microglia (Table 1). When MCP-1 was microcontact-printed with collagen, the number of ramified microglia as well as macrophagic microglia increased (Table 1). Microcontact printing of plasma induced microglial migration (Table 1). When plasma was spiked with MCP-1 and printed, the number of ramified microglia increased again (Table 1).

### 3.4. Epidemiology of the Patients in This Study

In the present study, 23 controls, 25 MCI, and 24 AD patients were included, maintaining a gender balance with 4–13 male subjects, and more females in the AD group (Table 2). The mean age was approximately 73 years and was significantly higher in the AD group (Table 2). The MMSE scores were 29.6 in controls, slightly decreased in MCI (27.4), and markedly decreased in AD (18.9; Table 2). The Geriatric Depression Scale score was 3.8 in controls and did not differ in MCI or AD groups (Table 2).

### 3.5. Effects of Plasma on Migration of Iba1-Positive Microglia

When plasma from control patients was printed, on average, 50 round Iba1-positive microglia, 12 ramified Iba1-positive microglia, and 12 Iba1-positive macrophagic microglia were found along the prints (Table 3). Plasma from MCI patients did not differ in these parameters (Table 3). However, plasma from AD patients significantly decreased the number of migrated round Iba1-positive microglia (12.9 ± 3, *p* = 0.01) compared to healthy controls (Table 3).

### 3.6. Mass Spectrometry

**Figure 4:**
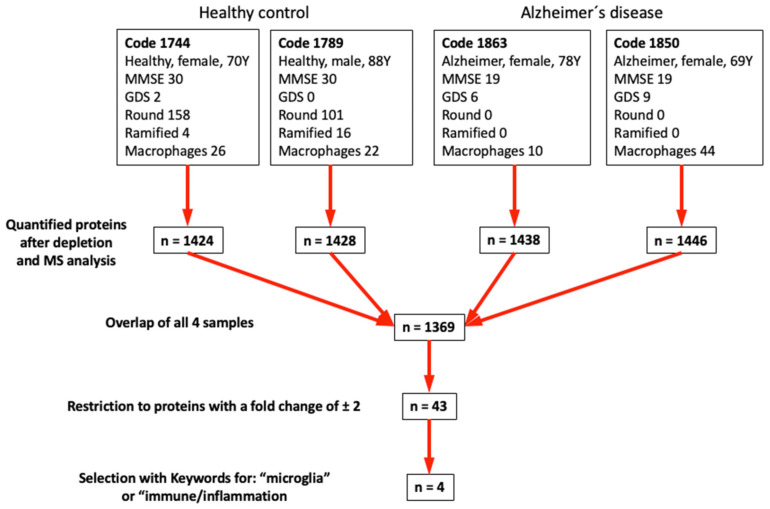
The four best plasma samples were selected (two controls and two Alzheimer’s disease) based on the experiments on activation of round microglia. After depletion of 14 proteins, the number of identified proteins is shown. Differential mass spectrometry and linking to the keywords “microglia” or “immune/inflammation” finally resulted in four identified putative biomarkers.

Based on the results in Table 3, we selected the top-two controls and top-two AD samples (Figure 4) and performed mass spectrometry. After depletion of the 14 most prominent proteins in plasma (e.g., albumin, immunoglobulins, fibrinogen, transferrin, etc.), we quantified approximately 1400 proteins in plasma, with an overlap of 1369 proteins (Figure 4).

Based on these results, we further restricted the number of proteins by the keywords “microglia” or “immune/inflammation”. This resulted in the identification of four putative biomarkers (Table 4): mannose-binding protein C, macrophage receptor MARCO, complement factor H-related protein-3, and C-reactive protein.

## 4. Discussion

The aim of the present study was to utilize mouse organotypic brain slices connected to µCPs loaded with human plasma. Following incubation, we observed activation and migration of Iba1-postive microglia along these µCPs. Our findings revealed that plasma from AD patients inhibited the migration of round microglia. Using the most promising samples, we identified four potential biomarkers: mannose-binding protein C, macrophage receptor MARCO, complement factor H-related protein-3, and C-reactive protein.

### 4.1. Iba1-Positive Microglia in Organotypic Brain Slices

Ex vivo organotypic brain slice cultures provide a unique method that combines the manipulability of in vitro models with the physiological integrity akin to in vivo systems. Unlike conventional in vitro cultures, organotypic brain slices maintain the integrity of all cell types in a three-dimensional slice, approximately 150 μm-thick, preserving both the cytoarchitecture and microenvironment [28,37]. Within our research group, these organotypic brain slice cultures are well established and extensively utilized [28], evidenced by over 60 publications. Commonly, brain slices are obtained from mouse donor animals at postnatal days 8–10 to ensure cell viability post-explantation, as they are more resistant to the mechanical trauma that occurs during brain slice preparation at this stage [38]. Brain tissue regions are cultured on a semipermeable membrane interface between a humidified atmosphere (maintained at 37 °C and 5% CO_2_) and the culture medium [39]. The brain slice flattens and become transparent during the two-week culture period, indicating high brain slice quality. Furthermore, this method facilitates the investigation of multiple variables within a single system, which can reduce variability across experimental setups. Organotypic brain slice cultures also align with the principles of 3Rs in animal research by decreasing the number of animals required and associated pain, as many slices can be obtained from a single brain.

Recent studies have demonstrated that microglia in brain slice cultures better preserve canonical microglia markers and overall gene expression compared to cultures of isolated adult microglia, a characteristic sustained for up to two weeks in vitro [40]. Analysis of global gene expression in human microglia revealed that human and mouse microglia share many similarities [41], particularly in how they adapt to environmental changes [42]. However, significant differences were noted in the context of aging [41]. Considering that we used postnatal organotypic brain slices, these differences were expected to be minimal. Iba1 (ionized calcium-binding adaptor molecule 1) serves as a widely utilized marker for microglia, enabling the detection and characterization of their morphology, including in organotypic brain slices [43]. Iba1 interacts with actin bundles, contributing to processes such as membrane ruffling and phagocytosis [44]. Unlike homeostatic microglial markers, such as transmembrane protein 119 (TMEM119) or P2RY12, Iba1 is also expressed by peripheral macrophages [45]. While Iba1 is commonly associated with microglial activation, it does not differentiate between functional microglial phenotypes. Nevertheless, Iba1 consistently stains diverse forms of microglia, encompassing ramified, amoeboid, and phagocytic types [45]. In our present study, we identified these three types of Iba1-positive migrated microglia: ramified microglia with numerous processes, amoeboid microglia characterized by their small, round morphology, and large macrophagic microglia. Ramified cells are typically considered involved in surveillance and synaptic pruning, whereas amoeboid and phagocytic microglia are often categorized as “activated”, engaging in phagocytosis and antigen presentation.

### 4.2. Microcontact Printing for Delivering Plasma to Brain Slices

In the past, various techniques have been utilized to deliver proteins, transfer genes, or transplant cells to brain slices, either via the culture medium or by adsorption onto different biomaterials. Recently, our lab has introduced and refined the microcontact printing technique to immobilize bioactive factors in µm-sized patterns, which we then connect to brain slices [29]. Due to its preciseness and ease of handling, microcontact printing is an appealing technique for guiding cells. We have successfully demonstrated the efficiency of microcontact printing in various contexts, including microglia [30], vascular structures [31], and nerve fibers [32], which underscores the robustness of our model. Microcontact printing, also known as soft lithography, uses a soft and, therefore, more biocompatible elastomeric stamp to transfer patterns of any protein onto a substrate [46]. However, it is important to consider that the plasma is immobilized through microcontact printing. Implementing a microfluidic system or further modifying the surface topography (e.g., creating microchannels) could improve the physiological aspects of the model [29].

Extracellular matrix substitutes, such as Matrigel, fibrin, polypeptides, hyaluronic acid, and collagen, are popular in three-dimensional cell cultures, with collagen being the most widely used [47]. Collagen, with its bioactive and biodegradable properties, provides both biochemical and physical guidance. A well-established delivery system is collagen hydrogel chemically crosslinked with four-arm PEG, which gradually releases the loaded biomolecule to the organotypic brain slice in culture [48]. Although collagen is typically found in the brain only in the basement membrane of vasculature, it is used as a biomaterial to enhance microglial motility and promote diverse microglial morphology [49,50]. This observation aligns with our findings, as microcontact printing of collagen alone enhanced microglial migration compared to sections without µCPs. We confirmed efficient printing by demonstrating a dose–response effect, achieving a minimal detectable protein concentration of 0.1 µg/mL of MCP-1. Additionally, we verified successful human plasma printing by visualizing MCP-1 spiked into the plasma samples.

### 4.3. Effects of Microcontact-Printed MCP-1 on Microglia

Chemokines are a family of small, conserved chemotactic cytokines that bind to G-protein-coupled receptors on the surface of cells, thereby regulating cell trafficking to sites of inflammation, damage, or trauma [51]. Chemokines and their receptors are expressed in endothelial cells, microglia, astrocytes, and neurons in the brain, and they have been shown to recruit distinct leukocyte subpopulations [52]. Microglia express several chemokine receptors, such as CC-chemokine receptor 2 (CCR2), with MCP-1 being the primary CCR2 chemokine ligand [53]. MCP-1 not only mediates microglial recruitment [54] but also induces their proliferation [55]. In AD, microglia play a dual role also in relation to the MCP-1 and CCR2 axis. Initially, microglia act protectively by accumulating via MCP-1 and eliminating toxic accumulations of Aβ, thereby slowing down AD progression [56]. However, when activated by MCP-1, microglia can contribute to AD pathology by releasing inflammatory factors, leading to neurodegeneration [54].

In our present study, we used MCP-1 as a control to demonstrate its effect on microglial migration by adding it to the medium or through microcontact printing. As hypothesized, the presence of MCP-1 in the medium significantly increased the number of migrated round microglia, ramified microglia, and macrophagic microglia. When MCP-1 was microcontact-printed with collagen, the number of migrated ramified and macrophagic microglia showed a significant increase. This study demonstrated for the second time [30] that microcontact-printed MCP-1 has the capacity to induce microglial migration, which can be efficiently evaluated.

### 4.4. Effects of Microcontact-Printed Plasma on Microglia

Cerebral amyloid angiopathy (CAA), characterized by small vessel disruption due to Aβ depositions, is frequently associated with AD [57]. Due to increased blood–brain barrier (BBB) permeability, microglia may be directly or indirectly affected by the extravasation of blood proteins [58]. At the brain vasculature, microglia play a critical role in maintaining a balance between protective and detrimental effects on BBB integrity [59]. The targeted migration of microglia toward injured or activated blood vessels is a protective mechanism, shielding the brain from neurotoxic factors. When activated, however, microglia can enhance BBB permeability and vascular leakage, potentially by releasing proinflammatory factors [59].

In our present study, we aimed to identify a blood plasma biomarker that induces neurotoxic microglial programming in AD. Interestingly, microcontact printing of healthy control plasma revealed a higher number of migrated round microglia than expected from the MCP-1 µCP results. However, plasma from AD patients significantly decreased the number of migrated round Iba1-positive microglia (*p* = 0.01) compared to healthy controls. This suggests the presence of a factor that inhibits microglial activation or migration, potentially driving microglia into a senescent or dystrophic-like state [18].

Recently, a research group identified fibrin as a blood protein inducing microglial polarization to neurodegenerative phenotypes [58]. We ourselves aimed to identify a set of potential biomarkers in plasma altering microglial migration in AD. So far, there are no plasma biomarkers available to diagnose AD in the clinics [60]. Plasma biomarkers would greatly simplify diagnosis concerning low costs, non-invasive intervention, and large-scale feasibility, enabling earlier symptomatic treatment options. Based on our findings of a lower migratory capacity along AD plasma µCPs, we performed mass spectrometry to identify factors responsible for the observed microglial senescence. We cannot rule out the possibility that microglial migration may be influenced by the age and sex of the plasma samples, as both are significant factors in AD. The prevalence of AD increases significantly with age, and females are more likely to develop AD than males [61]. To minimize the impact of these intrinsic variables, we included control samples from individuals above the average age and AD samples from those below the average age for mass spectrometry analysis.

### 4.5. Differential Mass Spectrometry

The improvement of new mass spectrometric methods, instrumentation, and bioinformatics tools led to the development of novel applications of mass-spectrometry-based proteomics. These advances have improved the application in clinical research, e.g., in the context of early disease diagnosis and characterization, biomarker discovery, and drug development. Proteomics methods are more and more preferred over conventional methods, mainly for their multiplexing capacity, remarkable analytical sensitivity and specificity, relatively shorter turnaround time, and potential for real-time in vivo analysis [62]. Further advances in mass spectrometry instrumentation will have a major impact in the field, with MS becoming a standard component of routine analysis and clinical practice.

Using differential mass spectrometry and proteomics, we identified four potential biomarkers for AD selective for the keywords “microglia” or “immune/inflammation”: mannose-binding protein C, macrophage receptor MARCO, complement factor H-related protein-3, and C-reactive protein. Using a PubMed search, we could find only one reference for mannose-binding protein C, that a mannose-binding lectin haplotype is associated with AD [63]. When linking the macrophage receptor MARCO and microglia, 23 references were found. This scavenger receptor mediates cytoskeleton rearrangements in microglia [64]. Silencing the MARCO gene resulted in a significantly reduced neuroinflammatory response [65]. No hits were found for complement factor H-related protein-3 regarding AD or microglia, while there is clear evidence that monomeric C-reactive protein plays a role in microglia and induces the cellular AD pathology [66]. Indeed, previous research has shown that elevated blood C-reactive protein is associated with increased AD risk in *APOE* ε4 allele carriers [67]. Out of these four markers, mannose-binding protein C seems to be the most promising, as it increased approximately 5-fold and has a mouse–human homology of over 60%. Mannose-binding protein C is a pattern recognition molecule of the innate immune system, involved in complement activation and neuroinflammation. Notably, one study found that mannose-binding protein C can bind Aβ [68]. While current evidence is limited [69], it is plausible to speculate that microglia modulate their dynamic functional phenotypes when exposed to mannose-binding protein C. Based on our results, we hypothesize that mannose-binding protein C inhibits the activation and migration of round microglia. The next steps to identify the potential role in AD will be: (1) We will develop a sensitive human ELISA to analyze the mannose-binding protein C in plasma, to determine the plasma levels and if there is a significant difference between healthy controls and AD. Additionally, we will test whether age and sex influence plasma levels of mannose-binding protein C. (2) Then, we will also generate recombinant human mannose-binding protein C and load these proteins into the µCP lanes to prove that, indeed, mouse microglia are influenced by these proteins.

### 4.6. Limitations of the Study

This study definitely has several limitations: (a) The µCP of plasma in collagen is not easy to perform and can vary between the different experiments. In order to rule this out, we prepared more prints, as necessary, and selected the best ones, as checked by the red-fluorescent control marker. (b) As the plasma was diluted in collagen and only an aliquot was printed on the membrane, we assumed that the amount of molecule on the print was rather low, and we expected a detection limit of approximately 10 ng/mL in plasma, which is far away from any sensitive ELISA detection system at the pg level. (c) Although brain slices from postnatal days 8–10 are commonly used for their enhanced cellular survival, they might not fully represent the complexity of neurodegenerative diseases seen in aging adults and AD. Unfortunately, the culturing of adult brain slices is not possible so far. (d) As we coupled mouse organotypic brain slices with human plasma, we assumed that any effect on the microglia must be induced by a mouse–human homolog factor. (e) Obtaining viable brain slices and keeping them alive in a culture environment is a method that requires skills acquired through experience. Although our team has extensive knowledge and experience in this method, determining criteria that prove that all brain slices have characteristics defined by strict standards will be an important factor for the future use of the method. (f) Although protein expressions provide very useful information about the function and pathology of specific cells in slices, the gene expression responses of these cells to different experimental applications will also be very important in terms of providing information that will support our hypotheses. We plan to use genetic analysis methods in future studies. (g) Although organotypic brain slice culture is one of the methods that best represents in vivo conditions, the fact that the slices are separated from the blood circulation and some axons are cut during slicing are factors that limit the similarity to in vivo conditions. (h) Thus, finally, the translatability of our findings to humans remains uncertain due to the complexity of the model, although this model may be very powerful to find immune-related factors that are altered in AD, specially using mass spectrometry. To date, there is no mouse model described that fully reflects all pathologies of human sporadic AD (Aβ plaques, tau/neurofibrillary tangles, neurodegeneration, vascular impairment, glial dysfunction, and inflammation) [70].

## 5. Conclusions

Taken together, we utilized mouse organotypic brain slices coupled with µCPs containing human plasma. After a two-week incubation period, we observed the activation and migration of Iba1-positive microglia along these µCPs. We found that plasma from AD patients inhibited the migration of round microglia. Using the optimal samples, we identified four novel biomarkers: mannose-binding protein C, macrophage receptor MARCO, complement factor H-related protein-3, and C-reactive protein. Our method represents a novel and innovative approach to translate research findings from mouse models to human applications.

## Figures and Tables

**Table 1:** Effects of MCP-1 on migration of mouse Iba1-positive microglia.

Treatment	*n*	Round	Ramified	Macrophagic	Length
Minus medium	5	0.2 ± 0.2 vs.	0.2 ± 0.2 vs.	0.8 ± 0.2 vs.	255 ± 89 vs.
MCP-1 medium	7	2.7 ± 1.7 **	6.7 ± 3.6 **	3.0 ± 0.7 **	364 ± 96
µCP (-)	5	5.8 ± 2.3 vs.	8.4 ± 3.3 vs.	7.0 ± 2.4 vs.	591 ± 76 vs.
µCP (MCP-1)	10	4.8 ± 1.7	28.2 ± 6.1 ***	14.0 ± 3.2 *	864 ± 26
µCP (Plasma)	6	13.1 ± 5.5 vs.	16.0 ± 5.9 vs.	16.0 ± 7.9 vs.	721 ± 63 vs.
µCP (Plasma+MCP-1)	10	11.3 ± 3.2	31.6 ± 6.2 **	9.4 ± 1.8	861 ± 63

Organotypic mouse brain slices were prepared and cultured without (minus) or with 100 ng/mL of monocyte chemoattractant protein (MCP-1) for 2 weeks. Alternatively, these slices were connected to microcontact prints (µCPs) loaded with PBS (-), MCP-1, human plasma, or plasma spiked with MCP-1. After 2 weeks in culture, slices were fixed, stained for Iba1, and counted. Values are presented as mean ± SEM, *n* is the number of independent animals, and the length is presented in µm. Statistical analysis was performed by one-way ANOVA with a subsequent Fisher LSD post hoc test (vs., versus; *, *p* < 0.05; **, *p* < 0.01; ***, *p* < 0.001).

**Table 2:** Epidemiology of patients who participated in this study.

Patient	*n*	Male	Age	MMSE	GDS
Control	23	13	73 ± 1.5 vs.	29.6 ± 0.1 vs.	3.8 ± 0.5 vs.
MCI	25	9	75 ± 1.6	27.4 ± 0.2 *	4.4 ± 0.6
AD	24	4	81 ± 1.5 **	18.9 ± 1.2 ***	5.0 ± 0.6

This table presents the patients included in the study, categorized into healthy controls, patients with mild cognitive impairment (MCI), and those with Alzheimer’s disease (AD). Mini-Mental State Examination (MMSE) scale and Geriatric Depression Scale (GDS) are stated Values are presented as mean ± SEM, *n* indicates the number of patients, and age is expressed in years. Statistical analysis was performed using one-way ANOVA followed by a Fisher LSD post hoc test compared to the controls (vs., versus; *, *p* < 0.05; **, *p* < 0.01; ***, *p* < 0.001).

**Table 3:** Effects of human plasma on migrated mouse brain Iba1-positive microglia.

Group	*n*	Round	Ramified	Macrophagic	Length
Control	23	49.6 ± 16 vs.	12.5 ± 1.9 vs.	12.5 ± 2.6 vs.	400 ± 50 vs.
MCI	25	27.1 ± 6	11.1 ± 2.6	10.5 ± 2.2	441 ± 51
AD	24	12.9 ± 3 *p* = 0.01 *	10.0 ± 2.2	13.1 ± 3.0	424 ± 55

This table presents the effects of plasma on migrated Iba1-positive microglia migrated on microcontact prints (µCPs), categorized into healthy controls, patients with mild cognitive impairment (MCI), and those with Alzheimer’s disease (AD). Values are presented as mean ± SEM, *n* indicates the number of patients, and the length is presented in µm. Statistical analysis was performed using one-way ANOVA followed by a Fisher LSD post hoc test compared to the controls (vs., versus; *, *p* < 0.05).

**Table 4:** Putative biomarkers identified by differential mass spectrometry and linked to the keywords microglia and/or immune or inflammation.

Putative Biomarker Identified	Symbol	Acc. No.	MW (kDa)	# Unique Peptides	Change in AD	Identity (%)	Similarity (%)
Mannose-binding protein C	MBL2	P11226	26.1	10	5.5 ↑	60.2	75.2
Macrophage receptor MARCO	MARCO	Q9UEW3	52.6	4	0.5 ↓	66.9	77.1
Complement factor H-related protein-3	CFHR3	Q02985	37.3	3	0.5 ↓	45.4	59.3
C-reactive protein	CRP	P02741	25	6	0.3 ↓	70.1	83

This table shows the gene symbol, the accession number in the data bank (Acc. No.), the molecular weight (MW), the number (#) of unique peptides, and the changes up or down in x-fold between controls and Alzheimer’s disease. Homology levels were calculated via NCBI BLAST+ and are presented as the percentage of characters that matched exactly (identity) and regions that may indicate functional, structural, or evolutionary relationships (similarity) between humans and mice.

## Data Availability

The data that support the findings of this study are available upon request from the corresponding author.
